# Incidence and mortality trends of nasopharynx cancer from 1990 to 2019 in China: an age-period-cohort analysis

**DOI:** 10.1186/s12889-022-13688-7

**Published:** 2022-07-15

**Authors:** Ruhai Bai, Jianzhong Sun, Yan Xu, Zhonghe Sun, Xiaoyan Zhao

**Affiliations:** 1 School of Public Affairs, Nanjing University of Science and Technology, Nanjing, China; 2 Health Science Center, Xi’an Jiaotong University, Xi’an, China; 3 Nanjing First Hospital, Nanjing Medical University, Nanjing, China; 4 Medical Innovation Research Division, Chinese PLA General Hospital, Beijing, China

**Keywords:** Nasopharynx cancer, Nasopharyngeal carcinoma, Incidence, Mortality, Age-period-cohort analysis

## Abstract

**Background:**

Nasopharynx cancer (NPC) is a great health burden in China. This study explored the long-term trends of NPC incidence and mortality in China.

**Methods:**

We retrospectively analyzed data from the Global Burden of Disease Study 2019 using an age-period-cohort framework.

**Results:**

The age-standardized incidence rate (ASIR) of NPC increased by 72.7% and age-standardized mortality rate (ASMR) of NPC decreased by 51.7% for both sexes between 1990 and 2019. For males, the local drift for incidence was higher than 0 (*P* < 0.05) in those aged 20 to 79 years. For females, the local drift was higher than 0 (*P* < 0.05) in those aged 30 to 59 years, and lower than 0 (*P* < 0.05) in those aged 65 to 84 years. The local drift for mortality rates were less than 0 (*P* < 0.05) in every age group for both sexes. The estimated period relative risks (RRs) for incidence of NPC were increased monotonically for males, and increased for females after 2000. The increasing trend of cohort RRs of incidence was ceased in recent birth cohorts. Both period and cohort effects of NPC mortality in China decreased monotonically.

**Conclusions:**

Over the last three decades, the ASMR and crude mortality rate (CMR) of NPC has decreased, but the ASIR and crude incidence rate (CIR) increased in China. Although the potential mortality risk of NPC decreased, the risk of NPC incidence was found to increase as the period move forward, and suggested that control and prevention efforts should be enhanced.

## Background

Nasopharynx cancer (NPC) is a squamous cell carcinoma that occurs in the intraepithelial mucosa of the nasopharynx and is characterized by distant metastasis. NPC has an extremely poor prognosis after metastasis, with a 91% fatality rate within 1 year after the initial metastasis [1]. Its local and lymph node recurrence and high risk of severe toxicity due to treatment measures also greatly complicate its prognosis [2]. According to the International Agency for Research on Cancer, NPC has a significant regional bias, with 129,000 new NPC cases worldwide in 2018, more than 70% of which occurred in Southeast and East Asia [3].

NPC is rare globally but common in China [4]. The age-standardized incidence rate (ASIR) of NPC in China was 3.0 per 100,000 in 2018, which is about 7 times higher than those found in mostly white populations [3]. Moreover, although previous studies have indicated that NPC incidence and mortality rates are declining worldwide [5], they are increasing in some regions of China. For example, the NPC incidence in Sihui county had an upward trend from 2003 to 2009, and in Cangwu county the NPC mortality rate increased from 1988 to 1998 and the incidence rate for males also increased slightly from 1983 to 2002 [6, 7]. As an area with high NPC incidence, China faces a huge burden.

Previous studies evaluated the incidence and mortality of NPC in China in specific year [4], and analyzed the long-time trends of diseases burden [6–8]. These studies provided important information on understand the health burden of NPC in China. However, few studies have explored changes in the incidence and mortality of NPC in different age groups in China. Moreover, the potential effects underlying the temporal trends nationally are still unknown. In this study, we used age-period-cohort (APC) frameworks to assess the long-term trends of incidence and mortality of NPC in China, and investigate the potential effects underlying these trends (age effect, period effect, and cohort effect). The results of this study provided necessary supplement information to the existing evidence on the burden of NPC in China, and provide a reference for allocating health resources and planning future health policies.

## Methods

### Data sources

This study used data sets from the 2019 Global Burden of Disease (GBD) study, which includes parameters such as morbidity and mortality for 369 diseases and injuries in 204 countries and territories between 1990 and 2019 [9]. For GBD 2019, the mortality data on China were mainly obtained from the Disease Surveillance Points and Vital Registration systems. Data on the incidence of NPC were obtained from individual population-based cancer registries and databases that include multiple registries. Data sources for the NPC incidence and mortality rates were extracted from the GBD database (http://ghdx.healthdata.org/gbd-2019). NPC incidence and mortality rates were standardized in this study using the global age-standardized population from the 2019 GBD study. In this study, NPC was confirmed based on International Classification of Diseases (ICD) diagnostic criteria (ICD10: C11-C11.9, D10.6; ICD9: 147–147.9, 210.7–210.9).

### Statistical analyses

We used an APC model to assess the trend of NPC incidence and mortality in China, and to assess the potential age, period, and cohort effects impact on these trends. The APC can be generally expressed as following [10]:

$$Y=\mathrm{log}(M)=\mu +{\alpha Age}_{i}+{\beta Period}_{i}+{\gamma Cohort}_{i}+\varepsilon$$

where $M$ for the incidence or mortality of the corresponding age group, $\alpha$ for the age effect, $\beta$ for the period coefficient, $\gamma$ for the cohort coefficient, $\mu$ for the intercept of the model, and $\varepsilon$ for the random error for the APC model. The problem of identifiability is the main objective problem in APC model, which is arises as an inherent mathematical relationship in the model. In order to overcome the issue of identifiability, we used weighted least squares regression to divide the effects of age, period and cohort, and then optimized the estimation of the three variables respectively [11].

The following parameters were evaluated in this study: net drift, which represents the overall annual percentage change; local drift, which represents the annual percentage change for each age group; the longitudinal age curve, which indicates the fitted longitudinal age-specific rates in the reference cohort after adjusting for period deviations; and the period (or cohort) relative risks (RRs), which is the RRs of a period (or cohort) relative to the reference period (or cohort) after adjusting for age and nonlinear cohort (or period) effects [12].

The APC model including arranging the data for incidence, mortality, and population into six consecutive 5-year time periods from 1990–1994 (median year = 1992) to 2015–2019 (median year = 2017). The entire population was divided into 14 age groups at 5-year intervals, from 20–24 years to 85–89 years. It was also divided into 19 consecutive birth cohorts at 5-year intervals, from 1903–1907 to 1993–1997. The web tool developed by the National Cancer Institute was used to obtain these estimable parameters [13]. In our APC model, the middle period (2000–2004) and cohort (1948–1952) groups were set as the reference groups. Wald chi-square tests were used to calculate the significance of functions and estimable parameters. All statistical tests were two-sided, and *P* < 0.05 was considered statistically significant.

## Results

### Incidence and mortality NPC trends in China from 1990 to 2019

**Fig. 1:**
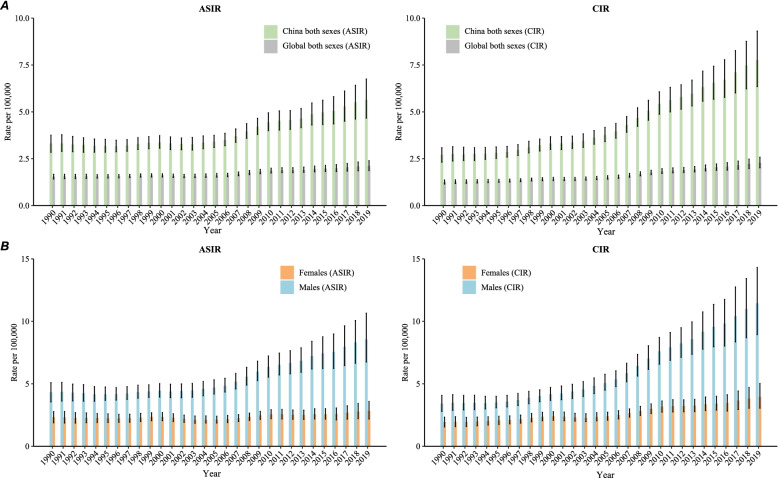
Age-standardized incidence rate (ASIR) and crude incidence rate (CIR) of Nasopharynx cancer in China, 1990 to 2019. **A** ASIR and CIR for both sexes, **B** ASIR and CIR for males and females. Age-standardized rates were standardized using the GBD 2019 global age-standard population

Figure 1A shows the NPC incidence trends in China from 1990 to 2019 for both sexes. In 1990, the ASIR of NPC was 3.3 per 100,000, and it increased to 5.7 per 100,000 in 2019 (increased by 72.7%). The crude incidence rate (CIR) in 1990 was 2.7, and it increased to 7.8 per 100,000 in 2019 (increased by 188.9%).

Figure 1B shows the trend in ASIR and CIR of NPC by sex in China from 1990 to 2019. For males, the ASIR increased from 4.3 per 100,000 to 8.6 per 100,000 (increased by 100.0%), and the CIR increased from 3.4 per 100,000 to 11.4 per 100,000 (increased by 235.3%). For females, the ASIR increased from 2.3 per 100,000 in 1990 to 2.8 per 100,000 in 2019 (increased by 21.7%), and the CIR of NPC increased from 1.9 per 100,000 in 1990 to 3.9 per 100,000 in 2019 (increased by 105.3%).

**Fig. 2:**
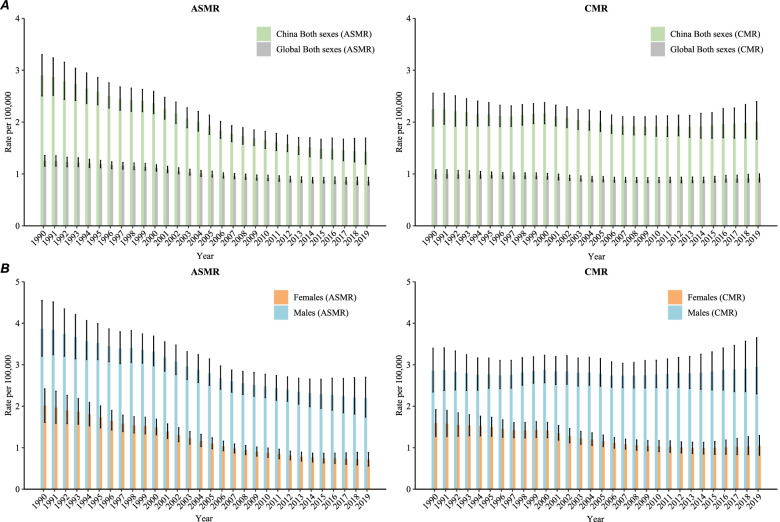
Age-standardized mortality rate (ASMR) and crude mortality rate (CMR) of Nasopharynx cancer in China, 1990 to 2019. **A** ASMR and CMR for both sexes, **B** ASMR and CMR for males and females. Age-standardized rates were standardized using the GBD 2019 global age-standard population

Figure 2A shows the mortality NPC trends in China from 1990 to 2019 for both sexes. In 1990, the age-standardized mortality rate (ASMR) of NPC was 2.9 per 100,000, and it decreased to 1.4 per 100,000 in 2019 (decreased by 51.7%). The crude mortality rate (CMR) in 1990 was 2.2, and it decreased to 2.0 per 100,000 in 2019 (decreased by 9.1%).

Figure 2B shows the trend in ASMR and CMR of NPC by sex in China from 1990 to 2019. For males, the ASMR decreased from 3.9 per 100,000 to 2.2 per 100,000 (decreased by 43.6%), and the CMR increased from 2.9 per 100,000 to 3.0 per 100,000 (increased by 3.4%). For females, the ASMR decreased from 2.0 per 100,000 in 1990 to 0.7 per 100,000 in 2019 (decreased by 65.0%), and the CMR of NPC decreased from 1.6 per 100,000 in 1990 to 1.0 per 100,000 in 2019 (decreased of 37.5%).

### Net drift and local drift for NPC incidence and mortality in China

**Fig. 3:**
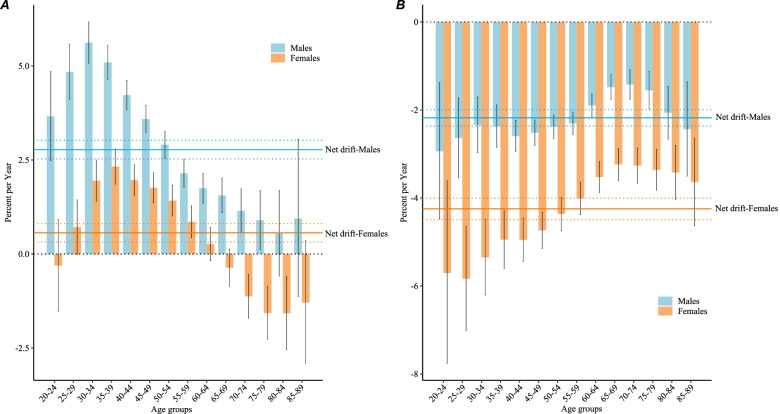
Net drift and local drift values for NPC incidence **A** and mortality **B** in China

Figure 3A shows the net drift and local drift values for NPC incidence in China. The net drift values of NPC incidence were 2.8% (95% confidence interval [CI] = 2.5% to 3.0%) for males and 0.6% (95% CI = 0.3% to 0.8%) for females. The local drift values of NPC incidence were greater than 0 in both males aged 20–79 years and females aged 30–59 years (*P* < 0.05). The largest local drift value for males was 5.6% (95% CI = 5.1% to 6.2%) in those aged 30–34 years, and for females it was 2.3% (95% CI = 1.9% to 2.8%) in those aged 35–39 years. The local drift of NPC incidence among females aged 65–84 years was less than 0 (*P* < 0.05).

Net drift (the overall annual percentage change), local drift (the annual percentage change for each age group), and the corresponding 95% CIs.

Figure 3B shows the net drift and local drift values of NPC mortality in China. The net drifts for NPC mortality were –2.2% (95% CI = –2.4% to –2.0%) and –4.2% (95% CI = –4.5% to –4.0%) in males and females, respectively. The local drift values for NPC mortality were less than 0 in every age group for both sexes (*P* < 0.05), indicating that NPC mortality has decreased for all ages over the past 30 years in China.

### Longitudinal age curves for NPC incidence and mortality in China

**Fig. 4:**
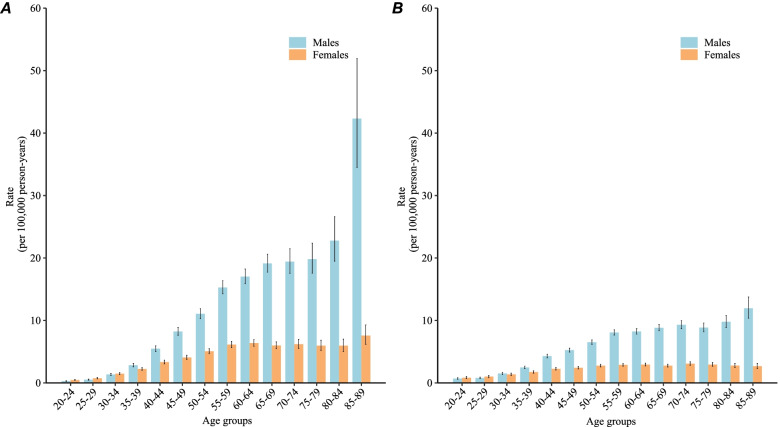
Fitted longitudinal age-specific rates of NPC incidence **A** and mortality **B** and the corresponding 95% CIs

Figure 4A shows the age-specific NPC incidence curves for China. For those younger than 30–34 years, the NPC incidence in the same birth cohort was similar between males and females, while the NPC incidence for those older than 39 years was significantly higher in males than in females (*P* < 0.05), with the curve significantly increasing with age.

Figure 4B shows the age-specific mortality curve for NPC in China. The trend was similar to that of incidence, and male mortality was significantly higher than female mortality after the age of 39 years (*P* < 0.05).

### Period and cohort RRs of NPC incidence and mortality in China

**Fig. 5:**
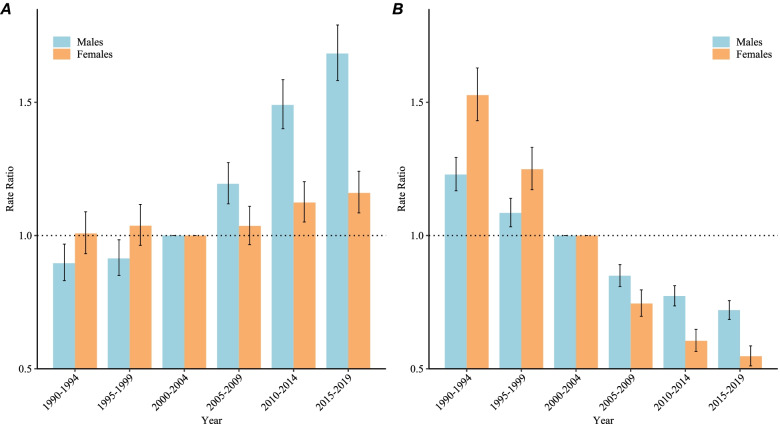
Period RRs of NPC incidence **A** and mortality **B** rates by sex in China

Figure 5A shows the period RRs of NPC incidence in China. The period RRs of the NPC incidence increased monotonically for males, and increased for females after 2000 compared with the reference period (2000–2004). Figure 5B shows the period RRs of NPC mortality in China, which indicate a monotonically decreasing pattern among males and females.

The RRs of each period compared with the reference period (2000 to 2004) after adjusting for age and nonlinear cohort effects, and the corresponding 95% CIs.

**Fig. 6:**
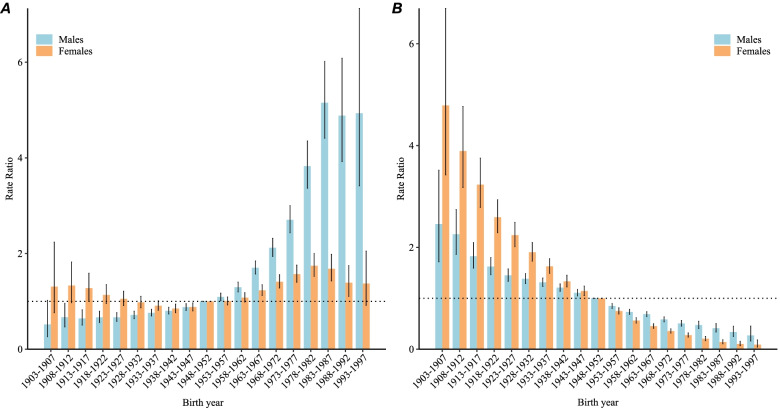
Cohort RRs of NPC incidence **A** and mortality **B** rates by sex in China

Figure 6A indicates that the cohort RRs of NPC incidence for males continually increased with each birth year, but stopped increasing in the recent birth cohort (1983–1997). For females, the NPC incidence increased after the 1938–1942 birth cohort, but this increase stopped after the 1978–1982 birth cohort. Figure 6B indicates that the cohort RRs of NPC mortality decreased monotonically for both males and females with birth years moving forward.

The RRs of each cohort compared with the reference period (1948 to 1952) after adjusting for age and nonlinear period effects, and the corresponding 95% CIs.

## Discussion

This study explored the long-term trend in the incidence and mortality of NPC in China, and detected the potential age, period, and cohort effects underlying these trends. The results of this study indicated that the ASMR and CMR of NPC in China decreased over the past 30 years. However, the ASIR and CIR of NPC increased. For each age groups, the incidence of NPC was decreased in elder females (65–84 years) over the last three decades, but increased in both younger females (30–59 years) and males (20–79 years). NPC mortality rates have decreased in all age groups for both sexes over the past 30 years. Although the period and cohort effects showed a monotonically decreasing pattern, the risk of NPC incidence increased as the period move forward.

Our study indicated that the CIR and ASIR of NPC increased over the past 30 years, which may be related to increased exposure to NPC risk factors, one of which is smoking [14]. China is the country with the largest tobacco consumption and production, with an estimated 316 million adult smokers, who account for nearly one-third of smokers and 40% of tobacco consumption worldwide [15]. Although the smoking rate in China has not changed much over the past 30 years, tobacco consumption has increased [16], with the sales volume of tobacco in China increasing from 76.92 billion to 127.48 billion packs from 2000 to 2014 [17]. Alcohol consumption is another important risk factor for NPC [18]. Consumption of alcoholic beverages has increased dramatically since the late 1970s in China, and high-risk drinking has reached epidemic proportions, with average annual per-capita alcohol consumption rising from 2.5 L in 1978 to 4.9 L in 2009 [19]. Meanwhile, more people participate in industrial production due to rapid economic development, which may increase occupational risks including dust and chemical smoke exposure, and may have also contributed to the increased NPC occurrence [20]. Previous studies have indicated that the number of workers exposed to silica dust in China increased from 12 million in 2004 to 23 million in 2009 [21, 22]. Furthermore, the increased NPC incidence in China may also be associated with the spread of PM2.5 [23]. Previous studies have indicated that salted fish (risk factor of NPC) consumption in China has increased, but these increases seem not to play an important role in explaining secular trends of NPC rates [24]. Increasing Epstein-Barr virus infection may be another possible reason for the increase in the incidence of NPC [25]. Family history also is an important risk factor for NPC, however, 30 years seems not enough to change this exposure.

A particularly interesting finding of this study was that although CIRs and ASIRs are increasing, the ASMRs in both sexes and the CMR in females have decreased in China, which may be related to a gradual improvements in prevention strategies and the recent significant progress in NPC diagnosis and treatment strategies [3]. In 2009, the Chinese Government committed to providing equal access to basic health care for all citizens and redirected significant resources to subsidizing primary health care facilities to ensure free preventive public health services for everyone [26]. Regarding screening methods, effective measures can identify patients earlier, resulting in a good treatment effect for early-stage (stages I and II) NPC patients, and may increase the 5-year survival rate to as high as 94% [27]. The current application of new technology significantly increases the coverage of early NPC screening over the historical cohort [28]. There has also been great progress in minimally invasive surgical techniques supported by endoscopy [29]. Moreover, induction chemotherapy plays a certain role in the local treatment of advanced NPC [30]. The present study found a difference between the CMR and ASMR, which may be related to the aging population of China, with the proportion of the elderly in the population rising from 7% in 1999 to 11.4% in 2017 [31]; elderly are a high-risk group for NPC, which increases its CMR. In our study, the incidence and mortality rate was not consistent with the previous study [32], which may be related to the use of different data sources. Compared with single data resources used in the previous study [32], GBD 2019 used the largest epidemiological and demographic datasets assembled to estimate incidence and mortality, which provides additional insight into the long-term burden of NPC.

In this study, the NPC incidence has the highest increase over the past 30 years among males aged 30–34 years and females aged 35–39 years, which was similar to the results of previous studies [33]. This may be related to smoking and occupational exposure. Those aged 30–34 years constitute the largest proportion of smokers, with not only a higher smoking rate but also being heavy smokers [34]. Moreover, this age group also had the highest NPC-related occupational exposure. These people are frequently exposed to pathogenic NPC factors such as smoke, dust, and chemical substances during their work. Multiple studies have found that occupational factors can increase the NPC risk by 2–6 times [35, 36]. Notably, NPC incidence in females aged 65–84 has decreased over the past 30 years, which may be related to improved lifestyles and an increased awareness of health and disease prevention [37].

Age is an important NPC risk factor. This study indicated that NPC incidence and mortality in males increases significantly with age, which is associated with accumulated exposure to risk factors [38]. Older people have higher NPC mortality rates than younger people may because of comorbidities and a poor health status that reduces their compliance with intensive therapy [39, 40]. This study also indicated that NPC incidence and mortality began to differ between males and females after 35–39 years old, with males having significantly higher values than females, which may be related to differences in lifestyle and career choices between the sexes. Males smoke and drink more than females [19, 41], and males were more likely to have jobs related to NPC exposure than females and hence increased occupational exposure to dust, physics and chemistry substances [42].

Period effects are factors that affect all individuals within a specific period [13]. Our study indicated that the RRs of NPC incidence increased monotonously in males over all periods, and increased in females after 2010. Regarding males, this may be related to their above-mentioned continuing increase in NPC risk factor exposure [20]. For females, although their exposure to risk factors such as smoking and drinking is lower than for males, exposure to NPC risk factors continues to increase with constant changes in Chinese culture and large improvements in the social economy. From 1991 to 2011, the average number of cigarettes smoked by females in China increased from 8.5 to 12.4 [43]. The average drinking rate among females in nine provinces in China was 9.7% from 1993 to 2006, and a nationwide study in 2012 indicated that the rate had increased to 13.9% and 13.3% in urban and rural areas, respectively [44], which may somewhat explain the recently increased NPC risk among females.

Cohort effects are the changes in disease risk from differences in risk factor exposure between birth cohorts [13]. The results of this study indicate that although the RRs of NPC incidence continually increased with later birth cohorts, the increase stopped after the 1985 birth cohort, and it decreased in females after the 1980 birth cohort. A possible reason for this is the regulatory policies on the tobacco industry in China. The Tobacco Monopoly Regulations legislation was issued by the State Council of China in 1983, and the state tobacco monopoly bureau implemented comprehensive tobacco controls to ban the production of substandard tobacco, reducing the scope and volume of inferior quality tobacco sales and reducing the tar and other harmful ingredients in tobacco that negatively impact the health of consumers and NPC risk. The period and cohort effects on NPC mortality continuously declined for both sexes in this study, which was related to the above-mentioned improvements in screening and treating NPC.

This study was subject to some limitations. First, the availability of primary data is the major limitation of GBD data sources [9]. Although GBD has already consolidated a large amount of primary data, the lack of more robust cause-specific mortality data in some remote and poor districts may affect the precision of GBD estimates. Second, it was an ecological study and inevitably had ecological fallacies, meaning that conclusions drawn from groups may not be applicable to individuals. To improve the understanding of the epidemic NPC trend in China, a large-scale cohort study should be conducted. Third, the theoretical basis behind APC model is complicated so that the actual meaning of parameter estimates could not be fully explained and the intrinsic meanings of the resulting of parameter estimates using this model are not intuitive. Fourth, the data in this study could not be used to analyze NPC trends for urban and rural regions of China. Due to recent rapid changes in rural life and work in China, the NPC prevalence may differ between rural and urban regions, and it is therefore necessary to explore these characteristics regarding NPC prevalence.

## Conclusion

Overall, this study indicates that the ASMR and CMR of NPC has decreased from 1990 to 2019, but the ASIR and CIR increased in China. By using the APC framework, we affirmed that the incidence of NPC increased among males aged 20–79 years, and females aged 30–59 years, but decreased among elderly females aged 65–84 years. The mortality rates were decreased in every age group. Although the potential mortality risk of NPC decreased in the different periods and birth cohorts, the risk of NPC incidence was found to increase as the period move forward. Given the ongoing aging process, NPC may have a huge impact on health in China in the future. This situation makes it necessary to identify NPC-related risk factors for prevention, to further develop prevention and treatment strategies, to focus on screening high-risk populations, and to implement measures for improving NPC diagnosis and treatment. Maximizing the accuracy of NPC staging and responsible allocation of public health resources to reduce the burden of NPC.

## Data Availability

The GBD 2019 data that support the findings of this study are available from the GBD Data Tool repository via the website of the Institute of Health Metrics and Evaluation (http://ghdx.healthdata.org/gbd-results-tool).

## Abbreviations

NPC: Nasopharynx cancer
APC: Age-period-cohort
GBD: Global Burden of Disease
ICD: International Classification of Diseases
RR: Relative risk
ASIR: Age-standardized incidence rate
ASMR: Age-standardized mortality rate
CIR: Crude incidence rate
CMR: Crude mortality rate
